# Development and Learner-Based Assessment of a Novel, Customized, 3D Printed Small Bowel Simulator for Hand-Sewn Anastomosis Training

**DOI:** 10.7759/cureus.20536

**Published:** 2021-12-20

**Authors:** Merieme Habti, Florence Bénard, Artur Arutiunian, Simon Bérubé, Dominic Cadoret, Léamarie Meloche-Dumas, Andrei Torres, Bill Kapralos, Frédéric Mercier, Adam Dubrowski, Erica Patocskai

**Affiliations:** 1 General Surgery, Université de Montréal, Montreal, CAN; 2 Health Sciences, maxSIMhealth Lab, Ontario Tech University, Oshawa, CAN; 3 Computer Science, maxSIMhealth Lab, Ontario Tech University, Oshawa, CAN; 4 Surgical Oncology, Centre Hospitalier de l'Université de Montréal (CHUM), Montreal, CAN

**Keywords:** simulator development, equipment design and procedural simulation, skills and simulation training, simulation trainer, realistic simulation

## Abstract

Hand-sewn bowel anastomosis (HSBA) is an essential skill for surgical residents to learn, as it is used in numerous surgical procedures. However, the opportunities to practice this skill before attempting it on patients are limited. Practice on simulators can help improve this technique, but there is a paucity of realistic, cost-efficient simulators for the acquisition of HSBA skills.

This technical report describes the development of our simulator that consists of a small bowel manufactured from silicone and a 3D-printed clamp system to hold the bowel in place. Our simulator was co-designed by a clinical team of surgeons and then assessed for perceived acceptability and effectiveness by 16 junior residents in various surgical specialties at our faculty. A majority of the learners rated our simulator to be a good or very good learning tool for HSBA, although they suggested some minor improvements.

Overall, our silicone small bowel model appears to be an effective and inexpensive way to acquire this surgical skill.

## Introduction

One of the fundamental skills learned by surgical residents is hand-sewn bowel anastomosis (HSBA). This skill is often used during colectomies or intestinal resections, and poor execution can lead to anastomosis leak, causing an array of complications ranging from abscess to peritonitis and septic shock [1]. Unfortunately, there are few opportunities for residents to practice HSBA before performing it in the operation room, with the attending surgeon supervising them.

Over the past few decades, fueled by increased attention to patient safety, reduction in residents working hours, and multiple handovers, modern surgical teaching methods showed a shift towards the use of simulator-based training (SBT). SBT has been shown to improve both surgical performance, technical skills, and even some evidence of improvement in teamwork and process outcomes [2,3]. Although a multitude of simulators has been developed and put on the market, costs and accessibility are considered barriers to the uptake of standardized SBT in surgical programs [4,5].

According to recent studies, 3D printing could enable the implementation of a more affordable and realistic SBT tool [6,7]. Indeed, many surgical specialties have developed and validated anatomical models to be used in their training curricula [8-11]. However, no 3D-printed simulation model has been designed specifically for HSBA.

This technical report aims to describe the development process of a 3D-printed, low-cost and realistic bowel model for HBSA training. To our knowledge, this is the first technical report outlining the process of co-designing such a simulator. We will describe the steps leading to the design of the HBSA simulator, in addition to the assessment of its perceived value as an educational tool (i.e., acceptability and effectiveness) by both experts and intended learners.

## Technical report

Context

This simulator was co-designed by two collaborative groups with complementary areas of expertise. Designers and graduate students from maxSIMhealth, a research laboratory located at the Ontario Tech University, Ontario, Canada (this group will be referred to as the development team), and a group of surgeons and surgical trainees located at the University of Montreal (this group will be referred to as the clinical team). The simulator was designed to train junior surgical residents in an at-home setting, as well as for standardized practices. The intended learners are residents who have no or very limited experience with the technique.

Inputs and design process

Design ideas for the simulator were initially discussed between the development and clinical team: with a senior researcher and a graduate student/designer as part of the development team and two surgical oncologists and two general surgery residents (junior and senior residents, respectively, who both had prior HSBA experience on in-vivo models and patients) as part of the clinical team.

The Architecture of the Simulator

All digital files of the anatomy were designed by the development team either based on publicly available medical illustrations or from digital models sourced from Creative Commons (Creative Commons, Mountain View, CA, USA) and subsequent feedback from the clinical team. The digital files were manipulated as a stereolithography (.stl) file in Fusion360™ (Autodesk Inc., San Rafael, CA). Using a secure digital (SD) card, the 3D-rendered files were transferred to an Ultimaker S5 3D printer (Ultimaker B.V., Utrecht, Netherlands) and were printed using white 3D-Fuel™ Pro polylactic acid (PLA) filament material (Fargo, ND).

**Figure 1:**
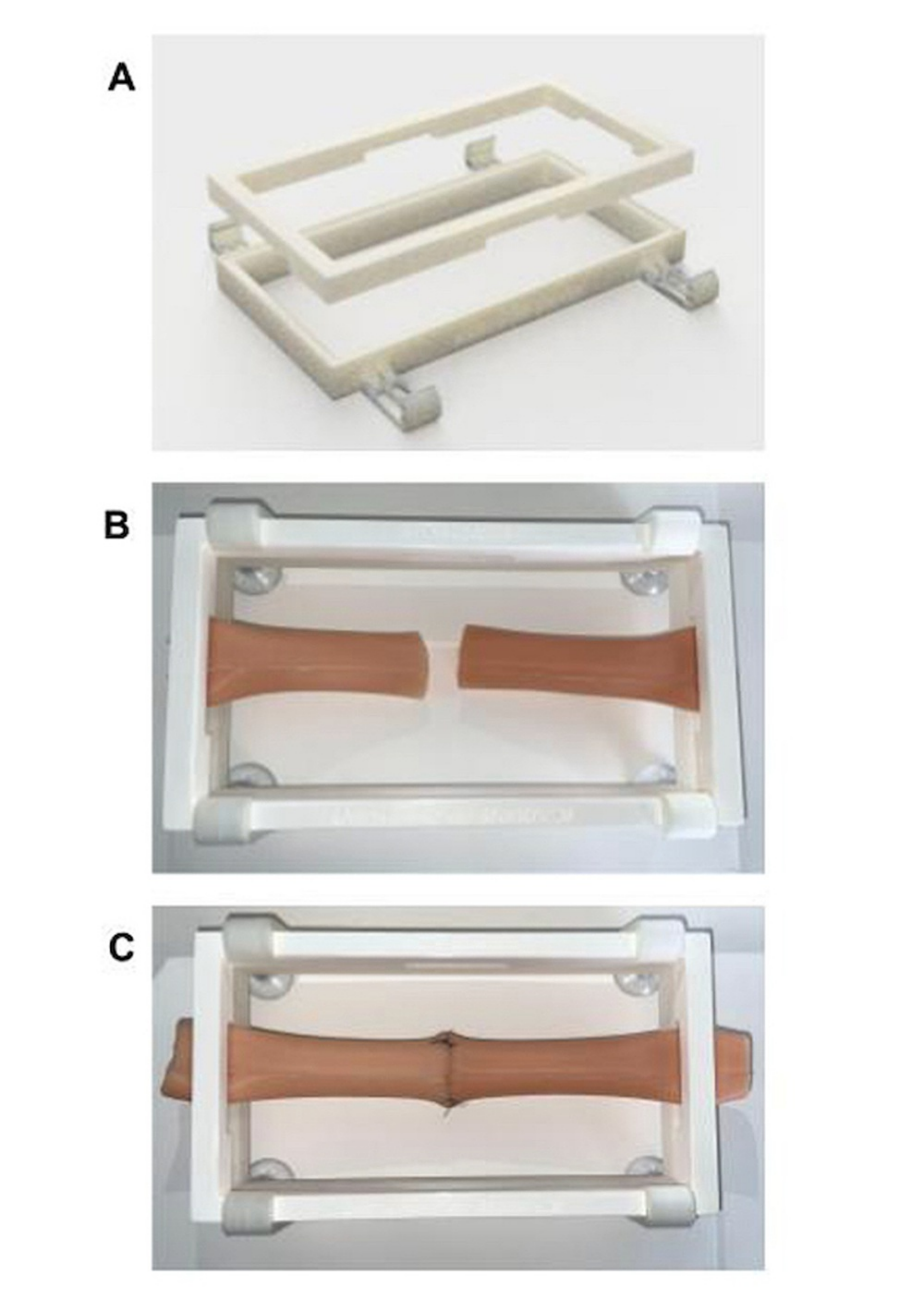
Simulator could be clamped to a tabletop Panel A shows the maxSIMclamp+ is separated into top and bottom parts. Panel B shows the HSBA silicone simulator is placed between the top, and panel C shows parts and then clamped using four latching-style clamps on the bottom part.

A stand (referred to as maxSIMclamp) previously developed for an Infant Intraosseous Infusion Simulator for Neonatal Resuscitation Simulator [12] was resized to fit the dimensions of the HSBA simulator, such that the simulator could be clamped to a tabletop (Figure 1). The stand was also uploaded to an Ultimaker S5 3D printer and printed using 3D-Fuel™ Pro PLA filament material (Fargo, ND).

All digital files that were developed for this project are shared publicly here: https://github.com/maxSIMhealth/UdeMHSBA. The use of all digital assets is bound by the terms and conditions of a Creative Commons Attribution-NonCommercial-ShareAlike 4.0 International Public License (CC BY-NC-SA 4.0). Subject to the terms and conditions of this Public License, the authors and creators hereby grant a worldwide, royalty-free, non-sublicensable, non-exclusive, irrevocable license to reproduce and share the licensed material, in whole or in part, for strictly non-commercial purposes; and produce, reproduce, and share adapted materials for non-commercial purposes only, under the same license. Please use this technical report as an acknowledgment for any research and description of educational activities that utilize any of these materials. 

Furthermore, the materials included in this technical report are the intellectual property of many individuals (e.g., students, clinicians, engineers, researchers). The creator (A Dubrowski) and his institution (Ontario Tech University) do not warrant that these materials are complete, true, accurate, or non-misleading. By using these materials, the user agrees that the exclusions and limitations of liability set out in this disclaimer are reasonable. If the user does not think they are reasonable, they must not use these materials.

Selection of Materials

**Figure 2:**
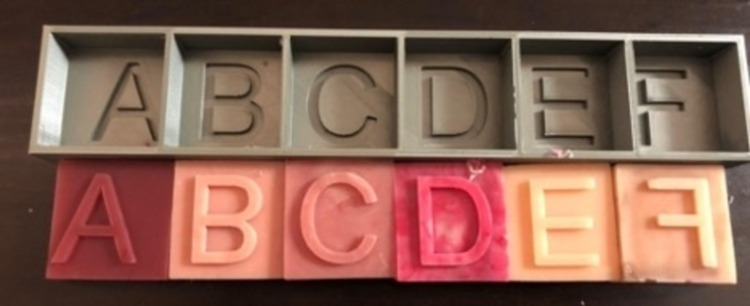
Silicone hardness and color samples The shore hardness of each sample was as follows: A=Shore 5A, B=Shore 00-30, C= Shore 00-10, D=Shore 10A, E=Shore 00-25, F=Shore 00-35 with nylon mesh.

Once the digital architecture of the simulator was identified, the teams discussed the desired texture, color, and thickness of the small bowel simulator. The development team built six silicone samples of varying attributes, some were more flexible while some were more rigid, and one had a mesh fabric included in it (Figure 2). The samples were letter-coded A-F to conceal the hardness and properties of each sample from evaluators. The letter-coding of samples was random and not in alphabetically ascending order. Two senior clinical team members tested all six samples separately, and both independently chose the same two models (B and E) as being most representative of the small bowel anastomosis tissue based on the desired texture, color, and thickness of the small bowel simulator. They also provided details regarding the required length, diameter, and thickness of the silicone small bowel simulator.

A small bowel simulator prototype was then produced by the development team and sent to the clinical team for testing with a survey via Google Form to provide feedback on the anatomical features, color, and haptic properties of the simulator, as well as list possible improvements. All clinicians confirmed that the prototype was representative of the small bowel anastomosis tissue, especially the replicated mucosae, and that it responded well to suturing. It was noted that knotting was different than in real tissues since the simulator had more recoil than a real bowel.

Assessment of the Simulator

Next, 16 first and second-year surgical residents from a variety of surgical specialties, including general surgery, urology, cardiac surgery, and obstetrics and gynecology, at the Université de Montréal were asked to provide feedback on the design of the simulator. To do this, 16 simulation kits, each including a 30-cm bowel model and a maxSIMclamp+, were manufactured at the maxSIMhealth laboratory. Half of the simulators (eight) were made available for testing at the local simulation laboratory, while the other half were sent to residents’ homes along with an instructional video through a gamified educational network (GEN) platform [13]. GEN provides several features to increase motivation, such as gamification elements and peer-to-peer collaboration. However, for this technical report, these features were disabled, and the GEN platform was used to provide interactive, on-demand instructions to the residents.

All residents subsequently provided feedback using an anonymous assessment survey adapted from the Michigan Standard Simulation Experience Scale (MiSSES) template for evaluation of simulation [14], which allowed us to assess their a) perceptions related to the simulator's representations of the anatomical features, b) the simulator’s potential to serve as an educational tool (i.e., perceived efficacy), and c) provide possible improvements to the simulator (Table 1). The survey included statements assessing for participant educational value of the simulator and the teaching quality of the simulation process. It also had open areas where participants could suggest possible improvements.

**Table 1:** Questions adapted from the MiSSES questionnaire. For the purpose of this technical report, we retained two of the original six MiSSES scales. As recommended by the developers, all answers were based on a 5-point scale with 1 denoting low levels of agreement and 5 high levels of agreement.

EDUCATIONAL VALUE
1. The simula(tor/tion) is a good training tool for knowledge in surgical techniques.
2. The simula(tor/tion) is a good training tool for skills in bowel anastomosis.
3. The simula(tor/tion) was critical at addressing bowel anastomosis techniques.
TEACHING QUALITY
4. Instructor(s) were knowledgeable about the topic.
5. Instructor(s) were able to convey material in a way that was understandable to me.
6. The learning materials (readings, presentations) improved my understanding of bowel anastomosis.
7. The resources we used improved my understanding of bowel anastomosis.
OPEN QUESTIONS/COMMENTS
8. Do you have any comments regarding the educational value of the simulator?
9. Do you have any comments regarding the teaching quality?
10. Please suggest any changes you would make to the simulator.
11. What specific changes would you suggest to improve your learning experience?

Products/Outcomes

Costs

Table 2 shows the breakdown of all costs associated with the manufacturing of the simulator. It is important to note that the mold for the bowel is a one-time cost. A single mold can be used multiple times to produce the silicone bowel simulator (each mold can produce up to four simulators). The maxSIMclamp+ is a part that can be 3D printed per individual user/station; therefore, it is also considered a one-time per-user cost. The silicone used to produce the small bowel is the only consumable material that needs to be accounted for. At this point, we do not have reliable data to provide an estimate of the number of sutures that a single simulator can tolerate. All cost estimates are in Canadian dollars (CAD), including local taxes, and these are based on the manufacturing cost of a batch of 18 simulators, which includes a single mold, 18 maxSIMclamp+, and 18 small bowel simulators.

**Table 2:** Cost breakdown of the materials (in CAD) needed to produce 18 HSBA simulators.

Material	Part	Amount	Cost (tax included)
3D printing material (Polylactic acid - PLA)	A single mold for 4 intestines (small bowel)	720 g	$21.60
	Clamp	160 g	$4.80
EcoFlex 00-30 Silicone	Intestineve (small bowel)	177 g	$8.00

*User A*ssessments

**Figure 3:**
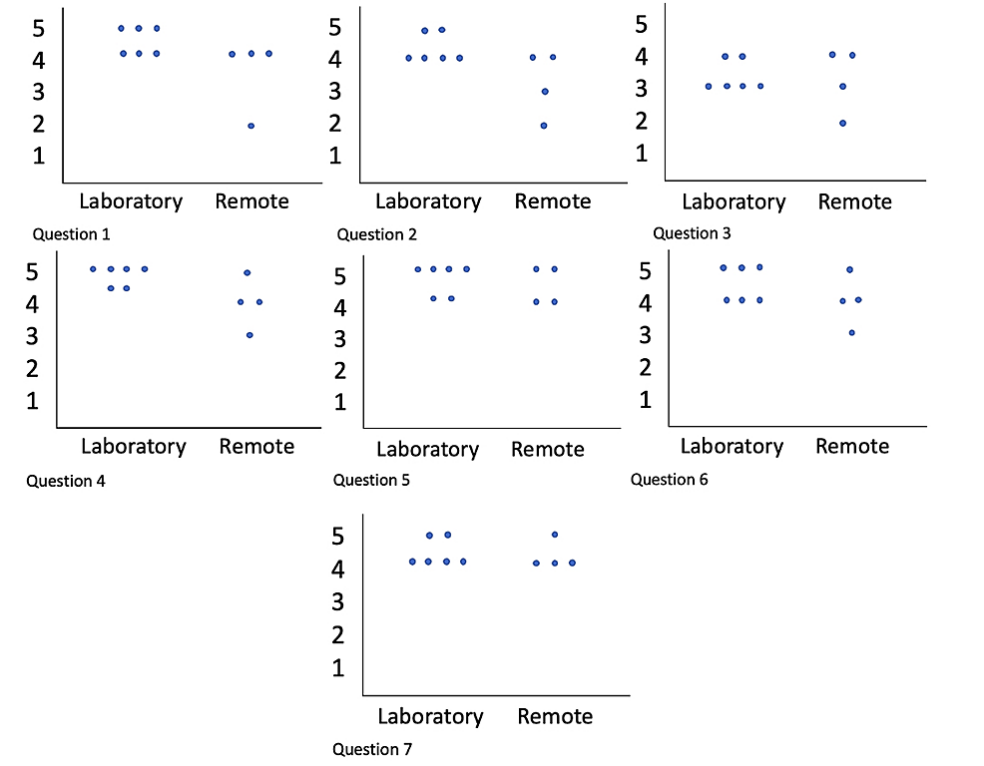
Scatter plot of the ratings for each of the questions. The plots illustrate responses (on a 5-point scale) to MiSSES questions corresponding to the questions illustrated in Table 1 and are expressed as a function of where the testing occurred: in the simulation laboratory vs. remotely, from the residents’ home.

Ten out of 16 residents completed the survey, for a response rate of 62.5%. Overall the majority of the residents rated the simulator to be good or very good to learn and practice skills related to HSBA (Figure 3). Most of them highly appreciated having such a tool at their disposal, although they mentioned they would like some minor improvements in the silicone’s texture. Because of the low response rate, we did not perform any inferential statistics on the data. Visual inspection of the plots suggests that there were no significant differences in the rating between the two types of the location of practice (in a simulation lab vs. remotely at home).

For questions 8 to 11 (Table 1), the participants in both groups' free-text comments are summarized in Table 3. In general, the simulator and the supportive instructional videos provided on the GEN platform were perceived as educationally useful, and the features of the simulators were perceived as realistic. It is important to note that the residents, especially those practicing remotely from home, perceived the instructional videos as a necessary component of the simulation. They all commented that the videos were helpful and well-matched the simulators. Few comments provided suggestions for improvements, such as improving the issue of ripping of the silicone material during suturing. To overcome this, we are currently testing embedding power mesh fabric (80% nylon/20% spandex; www.fabricland.com) during the silicone casting process.

**Table 3:** Summary of the free text comments.

Questions	Laboratory group	Remote group
Comments regarding education value	“Simulation was very good for familiarizing with steps of procedure, thus removing any hesitancy in what needed to be done. However, the model does not translate directly to true anastomosis given difference in material. Technique had to be modified while suturing silicone." “Definitely helpful to practice on the model beforehand.” “I believe this is a relevant addition to a resident's training, but the first few uses should be guided by a senior in order to correct our early mistakes.”	“Very interesting to have this training tool at our disposition for this type of technique” “Good simulation for this anastomosis technique. However, multiple different anastomosis techniques that are practiced in a clinical setting are not demonstrated through this simulation.” “The videos were useful.”
Comments regarding teaching quality	“Good learning opportunity.” “Great video, simple, easy to understand.”	“Instructional video was very clear and adapted to our level.” “Would possibly be useful to have other references on various anastomosis techniques +/- some information on when to use each technique.”
Please suggest any changes you would make to the simulator.	“The quality of the material (a lot of ripping) and the time allowed.” “The clamp mechanism should be reviewed because stability is a crucial element in the practice of the anastomosis considering the simulator consistency.” “No way of improving this, but silicone material for surgical technique is not representative of true bowel anastomosis e.g., suture bites had to be larger or else the silicone would rip.”	“A better way to display the simulator.” “The material was easily perforated, therefore significantly limiting its reusability (i.e., we had to cut a piece of the material off for each new anastomosis.)” “All the materials should be given with the simulator. The silicone material does not entirely resemble real life, which does not necessarily harm the process of learning the technique but could sometimes generate bad habits (taking larger bites while suturing to avoid the silicone ripping.)” “Make the intestine more user friendly.”
What specific changes would you suggest to improve your learning experience? Please use the space provided below to describe if needed.	“Have a new simulator for every test, have more time.” “Perhaps providing a video example of a bowel anastomosis performed on a true bowel, rather than just on the silicone model.”	“More training time between the pretest and the post-test.” “References on other anastomosis techniques.” “The video was good; the group was very well coordinated. I really appreciated their support.”

## Discussion

The main rationale for developing an inexpensive, customized HSBA simulator presented in this technical report is to provide more hands-on opportunities for junior residents to develop the technical skills needed to perform the skill in the operating room. Although most studies show no significant difference between HSBA and bowel anastomosis using surgical stapling, one study showed that residents had a higher incidence of anastomotic leakage with HSBA than more experienced surgeons [15,16]. Increasing resident training opportunities with our inexpensive, customizable simulator could potentially improve patient outcomes. Consequently, this report outlined the development of a realistic simulator for HSBA and the user-based feedback assessing the perceived anatomical realism and quality as an educational tool for surgical residents.

The costs related to the manufacturing of a single simulator were very low when compared to commercially available simulators. Specifically, based on the manufacturing cost of a batch of 18 simulators, which includes a single mold, 18 maxSIMclamp+, and 18 small bowel simulators, the costs of the first simulator are about 35.00 Canadian dollars. All subsequent simulators that utilize the same mold and maxSIMclamp+ cost only $8.00, while each maxSIMclamp+ costs $4.80, all prices in CAD. This significantly lower cost could make our simulator a good alternative for training surgical residents in underdeveloped countries.

After the initial use of the simulator by the residents practicing at the simulation laboratory and remotely from home, the simulator received overall positive feedback. They considered it to be a useful tool to learn and practice HSBA. Most of the residents who used the simulator in the simulation laboratory moderately or strongly agreed with the simulator allowing them to acquire both skill and knowledge in the HSBA technique in this setting. They appreciated being able to practice the technique without facing the pressure of being in the operating room, as the simulator allowed them to safely familiarize themselves with the steps of the procedure. Similarly, most of the residents who practiced remotely from home expressed similar assessments. Both groups of residents, however, commented that the simulator somewhat lacked realism, especially in its response to surgical instruments (i.e., ripped too easily during suturing). Specifically, since the silicone ripped too easily, they had to suture further from the bowel margins and secure their knots with lighter pressure, taking away some realism to the HSBA process. We are currently in the process of testing embedding power mesh fabric to overcome these issues. Similarly, the HSBA technique taught in the instructional video given to the residents had to be modified slightly to allow more distance between each suture, which some residents mentioned could lead them to develop bad surgical habits when performing an HSBA in the operating room. Despite these shortcomings, the majority of the resident respondents agreed or strongly agreed that the simulator was a useful tool to learn and practice the skill on and that the simulator increased their confidence level in performing the technique.

Participants in both groups also agreed or strongly agreed that the instructions and resources given on the GEN platform were useful and helped improve their understanding of the HSBA technique. One of them commented that they would have liked to practice other anastomosis techniques on the simulator since only one technique was taught to them. Some also mentioned that the clamp system could use some minor improvements, as the suction cups would not adhere to the table surface.

The main limitation of the user-based feedback was the limited experience of the residents since they were junior residents with little or no prior experience with the HSBA technique on an actual patient. That, in turn, potentially limited their ability to adequately assess the realism of the simulator. Their comments were relatively homogenous regarding the need to improve the texture of the silicone model but were not reflected by the clinical team since none of them had difficulty suturing the simulator in the development phase. Our development team is currently working on integrating a mesh fabric within the silicone to allow the surgical ties to be secured on the simulator without ripping through the silicone. This aims to provide a more realistic experience for the learner but implies a potentially higher production cost.

Based on a majority of the feedback provided by the junior residents, the simulator would need some minor adjustments before it can be considered for use in training. While it does reach its objective, which is to allow the learner to practice HSBA before having to perform it in a clinical setting, most respondents suggested minor improvements in the silicone texture to increase the simulator’s realism.

## Conclusions

Our simulator, combining a 3D-printed maxSIMclamp+ and a silicone simulator made from a 3D-printed small bowel mold, was demonstrated to be a cost-effective and useful tool in learning the HSBA technique in addition to being the first 3D-printed simulation tool designed specifically for HSBA to our knowledge. We believe our model, with a few minor improvements, could provide an inexpensive way for surgical residents to practice the HSBA technique, both in organized practice sessions and in home-based training sessions.
